# Morphology and development rate of the immature stages of Glyphidops (Oncopsia) flavifrons (Bigot, 1886) (Diptera, Neriidae) under natural conditions

**DOI:** 10.3897/zookeys.603.7355

**Published:** 2016-07-06

**Authors:** Andrés Felipe Vinasco Mondragón, Nancy Soraya Carrejo Gironza

**Affiliations:** 1 Departamento de Biología, Facultad de Ciencias Naturales y Exactas, Universidad del Valle, Santiago de Cali, Colombia

**Keywords:** Cactus flies, cephalopharyngeal skeleton, hypopharyngeal sclerite, immature stage, Nerioidea

## Abstract

Of the 116 Neriidae species known to date, 113 species have not been studied in their immature stages. Here, we examine the development of the immature stages of Glyphidops (Oncopsia) flavifrons (Bigot, 1886), which has one of the broadest distributions of Neriidae in southern North America, Central America, and South America; offering excellent opportunities for biological studies. A population of this species was monitored over a five month period. The following characteristics were tracked for a population located on the University of Valle campus in Cali, Colombia: oviposition duration, number of eggs per egg mass and lifespan of each immature stage (egg, larva, and puparium) under natural conditions (*in situ*). The external morphology of the egg, larva, and puparium were described; their stages lasted 58 (± 4) hours, 10 (± 1) days and 13 (± 1) days, respectively. The lapse of time for each larval instar was statistically supported by using Tukey comparisons and cluster analysis of hypopharyngeal sclerite length and mandibular area. In addition, it was also sustained throughout the morphological study of structural changes in mouth hook, and anterior and posterior spiracles. Finally, the presence of the labial and epipharyngeal sclerites are reported as new characters of Nerioidea. Natural history data are provided.

## Introduction

Neriidae (Diptera: Brachycera) is represented by 116 species grouped in 17 genera (Pape et al. 2011, Sepúlveda et al. 2014). Studies of this family in the Neotropical Region have increased in the past five years, focusing on its adult stage (Sepúlveda et al. 2013a, 2013b, 2014, Mongiardino et al. 2014). However, since 1947, only three species from all around the world have been described in their immature stage (Berg 1947, Olsen and Rickman 1963, Mangan and Baldwin 1986). *Telostylinus lineolatus* (Wiedemann, 1830), an Australo-Oceanic species, whose larvae were described from eight mature larvae and 12 pupae collected on the banks of the Tenaru River in the Solomon Islands and studied by Berg (1947). *Odontoloxozus longicornis* (Coquillett, 1904), distributed from the southwestern United States to Costa Rica (Olsen and Rickman 1963, Mangan and Baldwin 1986), was described based on larvae raised for several generations in necrotic tissue of *Opuntia occidentalis* Engelm. from San Dimas Canyon, California by Olsen and Rickman (1963). *Odontoloxozus pachycericola* Mangan & Baldwin, 1986, was studied from senita cactus (*Lophocerus schottii* (Engelm)) and cactus carbon (*Pachycereus pringlei* (S. Wats.) from the cape region of Baja California, México and bred for several generations by Mangan and Baldwin (1986). The study, however, focused on the adult and only the number of pro-thoracic spiracular papillae of the 3^rd^ larval instar was determined. Neverthless, Foote and Teskey (1991) proposed that neriid larvae lack diagnostic distinctive characters that allow them to be properly separated from other muscomorphan saprophagous families like Micropezidae or Cypselosomatidae (McAlpine 1989, Wiegmann et al. 2011).

Regarding their biology, some authors consider neriids as synanthropic or at least opportunistic flies (Barraclough 1993). Eberhard (1998) taped adults of *Glyphidops flavifrons* and *Nerius plurivitatus* displaying aggressive behavior, copulation, and oviposition over branches of a fallen tree on a decomposition stage in a mature rainforest in Panama. In the same country, Cresson (1938) observed neriid adults on decomposing flesh of *Cereus* Mill., pumpkin (*Cucurbita* L.) and rotting trunks of papaya (*Carica papaya* L.). In North America, Olsen and Ryckman (1963) found and bred *Odontoloxozus longicornis* (Coquillet) larvae from eggs laid in necrotic tissue of several cactus species and Steyskal (1987) reported it on stems of *Carica papaya*. Preston-Mafham (2001) reports males of *Gymnonerius fuscus* and *Telostylinus* sp. guarding rot-holes (beetle larval borings and female oviposition in fallen Mango branches in Sulawesi, Indonesia. Barraclough (1993) reports *Chaetonerius* larvae reared from decaying pumpkin in South Africa and Zimbabwe and also proposed that *Chaetonerius apicalis* could develop in fruits or flowers of *Strelitzia nicolai* Regel & Koern. Finally, Bezzi (1928) cited by Berg (1947) reported neriid larvae in cotton capsules from Australia.

Glyphidops (Oncopsia) flavifrons (Bigot, 1886) can be found throughout the Neotropical Region, from south-eastern Brazil (Espirito Santo) to the southern United States (Arizona, Florida) in the southern Nearctic Region (Sepúlveda et al. 2014). Its reproductive behavior has been studied by Eberhard (1998), yet their immature stages remain unknown. The present paper will describe the immature stages and life history of Glyphidops (Oncopsia) flavifrons and report development time for each life stage under natural conditions in Cali, Colombia, during May and April (2014). Larvae of Glyphidops (Oncopsia) flavifrons are compared morphologically with those of *Odontoloxozus longicornis*, *Odontoloxozus pachycericola*, and *Telostylinus lineolatus*.

## Materials and methods

### Breeding and immature lifespan

*Glyphidops flavifrons* was reared *in situ*, between the months of March and May, 2014 on the Melendez campus of the University of Valle located in Santiago de Cali, Colombia (3°22.448'N; 76°32.084'W; 987 masl) found in the tropical dry forest life zone *sensu* Holdridge (1967).

The study area was composed of *Carica papaya* trees and *Tradescantia zebrina*, with vegetation coverage varying between 58% and 70%.

For the breeding process, fresh *Carica papaya* stems were cut into 30 cm long pieces and placed at the study site in a plastic container to protect them from other organism during decomposition (2–3 days). Afterwards the stems were exposed to adults of Glyphidops (Oncopsia) flavifrons population (previously identified) for four hours. The time of oviposition was recorded. The egg masses were individualized by one-ounce plastic containers with fragments of *Carica papaya* of 8 mm, each container was labeled and covered with fine mesh to allow ventilation and prevent intrusion by other invertebrates. To prevent injury of the eggs, the number of eggs per egg mass was recorded after the maturation of it (24 hours later) (Olsen and Rickman 1963, Craig 1967). After hatching, the larvae were observed daily. The puparia were individualized in plastic containers containing a layer of sifted and sterilized soil.

For the developmental rate assessment, 30 eggs were separated and observed every four hours until hatching. After hatching, ten larvae were sacrificed, following the method proposed by Adams and Hall (2003). Ten larvae were sacrificed daily until pupation occurred. An observation of 15 puparia was made every 12 hours, until emergence.

Humidity and temperature data were compiled daily, at 15 minute intervals with the help of a Dickson Data Logger TP125.

### Morphology

**Egg**

Twenty-six eggs were set on hollow plates with distilled water. Polar diameter and respiratory filament length were measured. Description follows the terminology used by Olsen and Rickman (1963).

**Larva**

To ensure accuracy, larval body length was measured immediately after sacrifice (Adams and Hall 2003). Micro-preparation of cuticular surface and cephalopharyngeal skeleton was performed following the methodology suggested by Niederegger et al. (2011). Body length (lateral view), hypopharyngeal sclerite length, and mandibular area (mandibular sclerite + mouth hook) measurements were performed daily. Additionally, antennal variations, maxillary palp, antenomaxillary lobe, and spinulose areas were observed. Description follows the terminology used by Foote and Teskey (1991).

**Puparium**

Total length of puparium was measured and morphology of both anterior spiracles (prothoracic spiracle) and thorny areas were examined. To determine duration of pupariation, photographic records were performed every 15 minutes for 150 minutes after pupation initiated (Bunchu et al. 2012).

### Measurements and images

Measurements of egg, larva, and puparium were performed using tpsDig2, version 2.22 (Rohlf 2007).

Photographic records were performed using a Canon EOS Rebel T3i camera, adapted to a Nikon Eclipse E200 microscope. Photographic compilation was done using Helicon Focus software. Diagrams were constructed with Corel DRAW program.

### Data analysis

Larval instars were determined by using a one-way ANOVA (confidence level: 95%). Post-ANOVA (Tukey comparisons) was used to calculate the variation of hypopharyngeal sclerite length and mandibular area throughout the observation period. Moreover, a cluster analysis was performed for each measurement (including body length) using Euclidean measures and neighbor-joining as a linkage method for each cluster.

Additionally, scatter plots were graphed for each of the measured variables to monitor their distribution over time. Box-plot graphs were used to compare distribution between the variables for each different larval instar (Ln). Figures and analyses were performed using Microsoft Excel 2013 and Minitab16 software.

## Results

### Development time

Development time of Glyphidops (Oncopsia) flavifrons was determined under natural conditions, temperature mean 25.8 °C (maxim. 41.06 °C, minim. 18.6 °C) and relative humidity mean 69.38% (maxim. 82.9%, minim. 44.9%). The eggs hatched 58 ± 4 hours (n = 30) after being laid. The total larval development time was 10 ± 1 days (n = 15) and the puparium stage had a development time of 13 ± 2 days (n = 15).

ANOVA showed significant differences for values of mandibular area versus time (df = 9.40; F = 1829.61 and P = 0.000) and the hypopharyngeal sclerite length versus time (df = 9.40; F = 7870.85 and P = 0.000). Post-ANOVA of mandibular area showed four groups, and post-ANOVA of hypopharyngeal sclerite length showed three groups, thus confirming three distinct larval instars L_1_, L_2_ and L_3_. Life spans of all larval instars are summarized in Table 1.

**Table 1.** Size and life span for each Glyphidops (Oncopsia) flavifrons larval instar, under natural conditions (25.79 ± 4.11 °C, 69.38 ± 9.23 % H.R.

Post-hatching days	N° larvae	Body length (mm)	Mand. Area (mm^2^)	Post-ANOVA mand. area †	Hypophr. Scl. Length (mm)	Post-ANOVA Hypophr. Scl. length †
**L_1_**						
**1**	10	1.22–1.75	0.00084	A	0.097	A
**2**	8	1.88–2.52	0.00085	A	0.1	A
**L_2_**						
**3**	11	2.95–4.45	0.0045	B	0.13	B
**4**	11	4.41–6.2	0.0047	B	0.13	B
**L_3_**						
**5**	6	6–9.56	0.0134	C	0.24	C
**6**	7	6.58–10.53	0.015	D	0.24	C
**7**	10	6.35–10	0.015	D	0.24	C
**8**	10	7.99–10.59	0.015	D	0.24	C
**9**	11	8.87–11.16	0.015	D	0.24	C
**10**	7	8.14–10.08	0.015	D	0.23	C
**Instar summary**						
**L_1_**	18	1.76 +/- 0.12	0.00084		0.098	
**L_2_**	22	4.36 +/- 1.14	0.0046		0.13	
**L_3_**	51	9.17 +/- 1.53	0.015		0.238	

† Post-ANOVA individual confidence level: 99.82%

**Figure 1.**
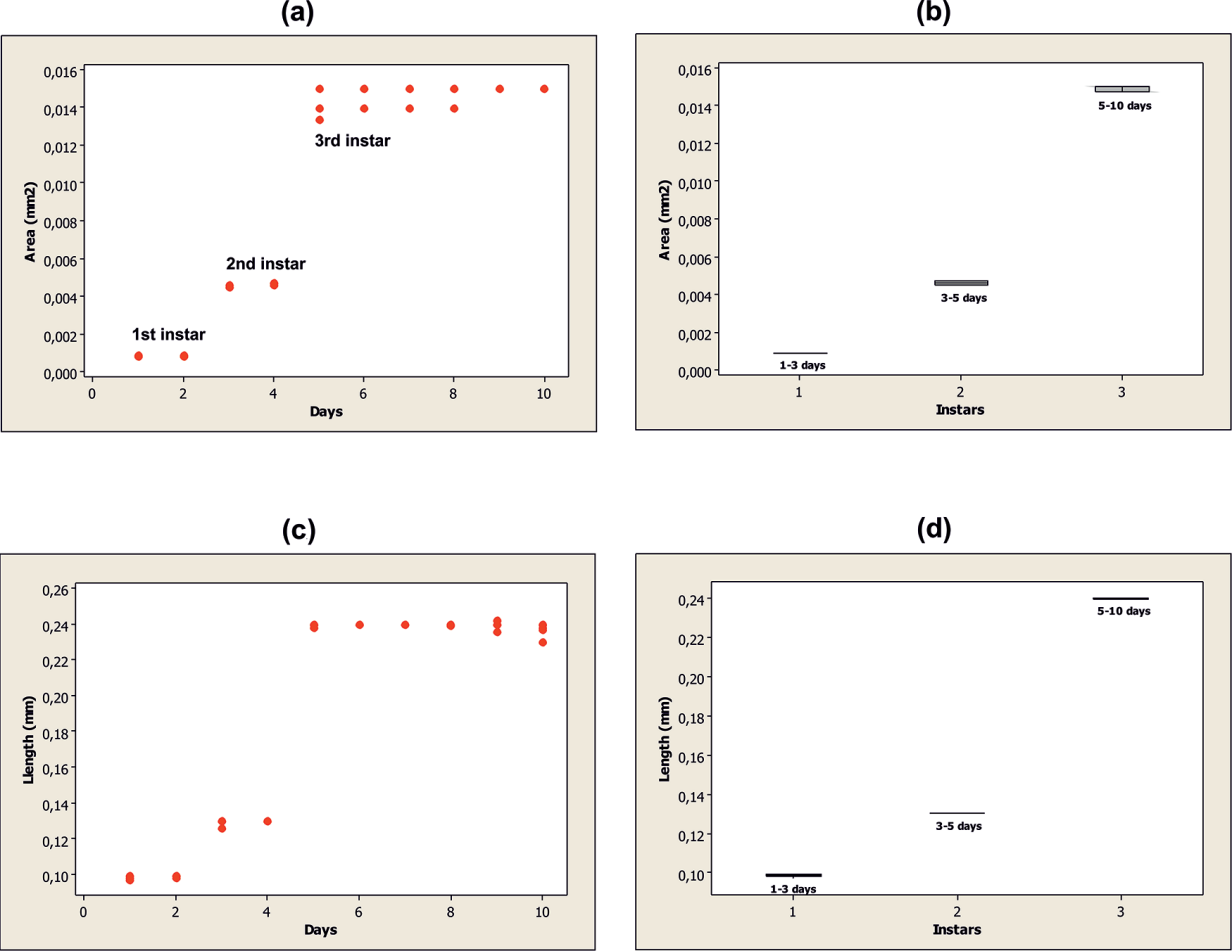
Scatterplot and Box-Plot: **a, b** mandibular area **c, d** hypopharyngeal sclerite length.

Scatter plots (Fig. 1a and c) show two distinct jumps in growth for the observed structures: the first between day 2 and 3 and the second between day 4 and 5. Furthermore, box-plot graphs (Fig. 1b and d) graphically support the findings from the Tukey test, by illustrating the variation in each structure’s measurements.

### Morphology

**Egg**

**Figure 2.**
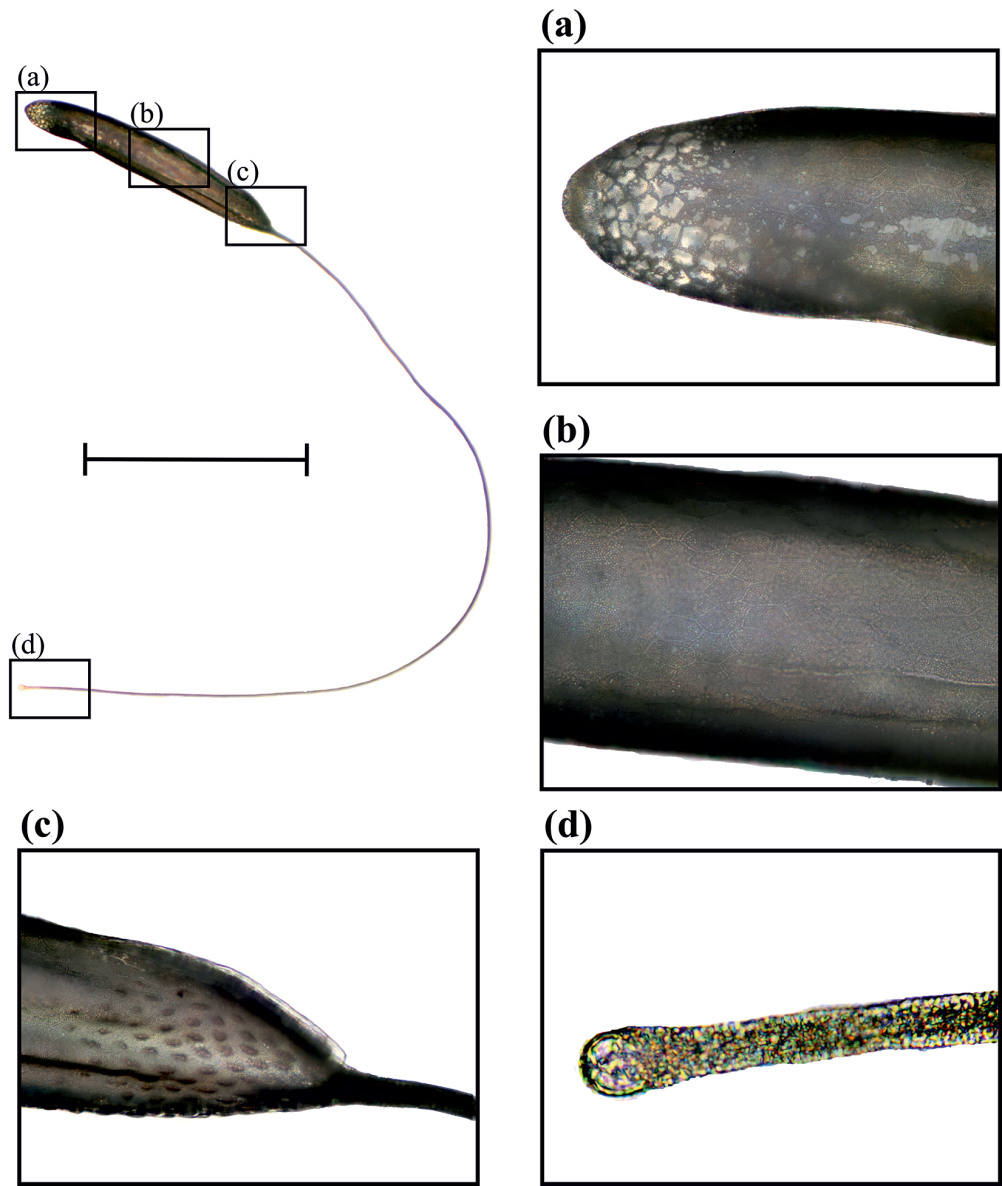
Egg in latero-dorsal view. Details **a** posterior end **b** average area of the egg, note the hexagonal pattern of the corium **c** basal respiratory filament point, note the tubercles over the apical area of the egg body **d** apex of the respiratory filament. Scale bar: 1 mm.

Body length 1–1.24 mm (x = 1.16, n = 20); respiratory filament length 3.13–4.01 mm (x = 3.3; n = 20). Body semi-cylindrical, dorsally convex and dorso-ventrally flattened, with a blunt posterior region. Respiratory filament originates from the anterior region, as long as 3.21 times the egg body length (Fig. 2). Two longitudinally lateral hatching lines departing from the respiratory filament attachment point and dim gradually towards the 1/6th posterior region of the egg body. Chorion sculpted with cells varying from pentagonal to octagonal (Fig. 2b), forming a mesh-like pattern that is more conspicuous in the posterior 1/6th. Anterior quarter region of the egg body with a small elliptical tubercle band visible with 10× magnification (Fig. 2c).

**Larva**

**Figure 3.**
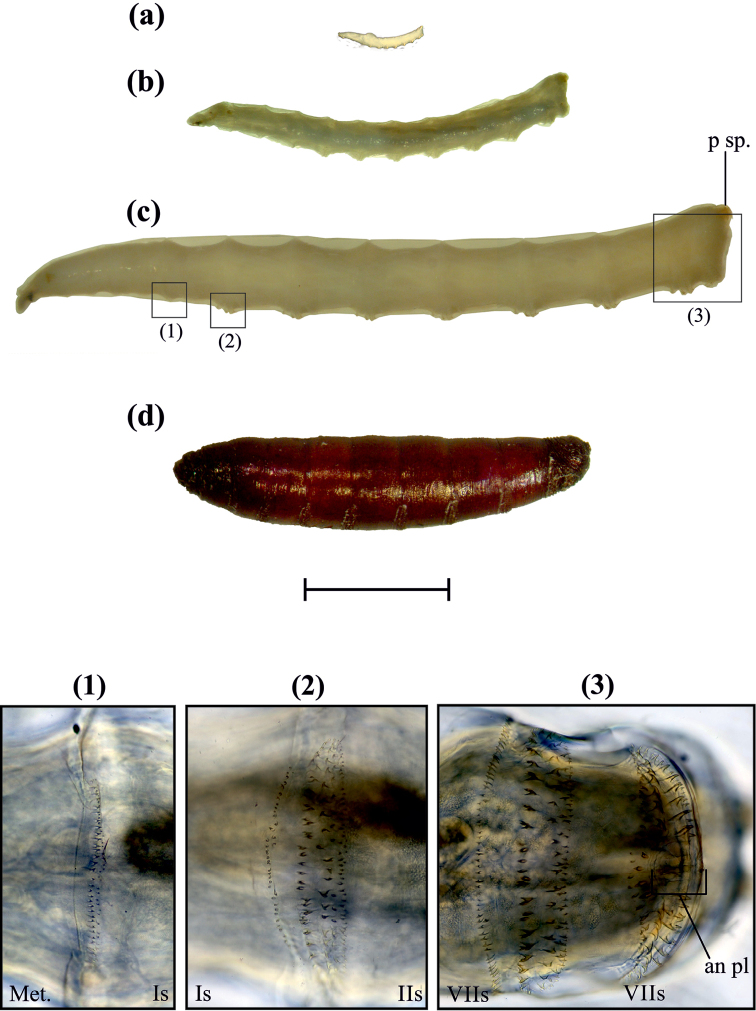
Larvae and puparium in lateral view and approach to the third larval instar spinulose areas. **a** L_1_, **b** L_2_ and **c** L_3_ **d** Puparium. (1) Spinulose area of the first abdominal segment, (2) posterior row of spines on abdominal segment I and anterior spinulose area on abdominal segment II, (3) posterior row of spines on segment VII and spinulose areas on abdominal segment VIII. an. pl., anal plate; p sp, posterior spiracles; Met. metathorax. Scale bar: 2 mm.

**Figure 4.**
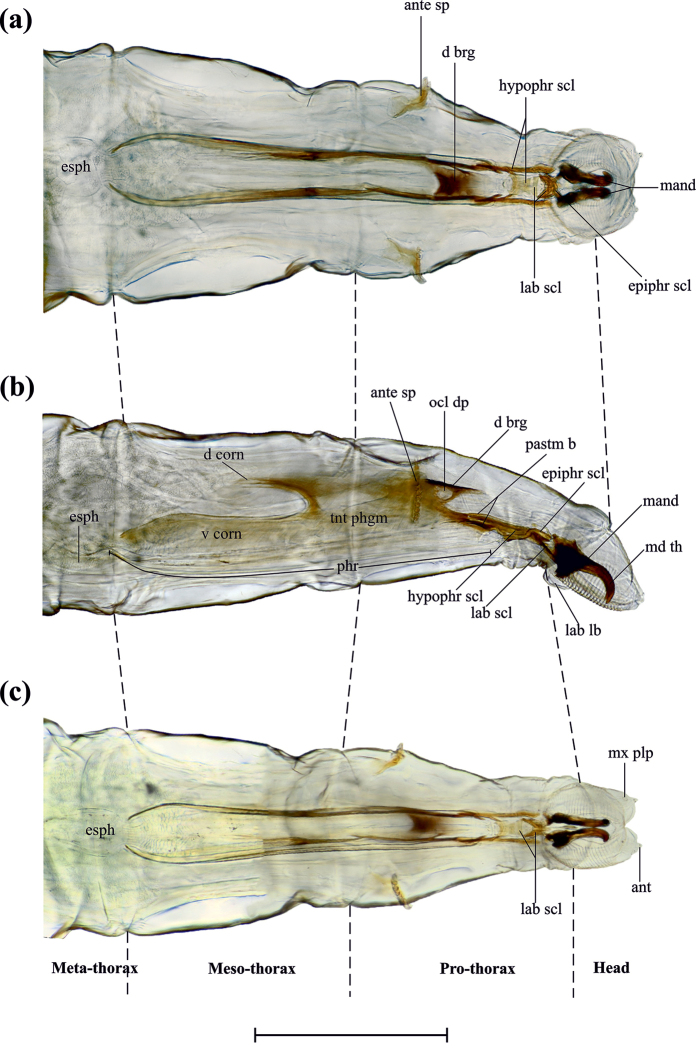
Cephalopharyngeal skeleton. **a** dorsal view **b** side view **c** ventral view. ant, antenna.; ante sp, anterior spiracle; d brg., dorsal bridge; d corn, dorsal cornu; epiphr scl, epipharyngeal sclerite; esph, esophagus; hypophr scl, hypopharyngeal sclerite; lab lb, labial lobe; lab scl, labial sclerite; mand, mandibule; md th, mandibular tooth; mx plp, maxilar palp; pastm b, parastomal bar; phr, pharynx; ocl dp, ocular depression; tnt phgm, tentorial phragm; v corn, ventral corn. Scale bar: 3 mm.

Vermiform body, glabrous, light to semitransparent (Fig. 3a–c). Head: (Fig. 4a–c) retractable, with two pairs of appendages located antero-dorsally; maxillary palpus bulbous, with an apical sensory depression, containing four to five tubercules with bristles over them; antenna reduced, with two to three antennomeres; a pair of ventrally curved mouth hooks, each originating from a mandibular sclerite (Fig. 6a–c.); antenomaxillary strongly lobed: lobes with 28–30 oral or pseudo-tracheal bridges, each radiating from the labial lobe; epipharyngeal sclerite (Fig. 4a) U-shaped with projections joining ventro-posteriorly to the back margin of labial sclerite; labial sclerite (Fig. 4b and c), arrow-shaped, directed antero-ventrally; Thorax: Pro-, meso-, and metathorax well-defined. Hypopharyngeal sclerite H-shaped, as long as 5.5 times its width (lateral view), formed by two parallel bars connected by a strong bridge originated in the anterior half of the sclerite, “bridge” concave, forming a canal that links antero-dorsally with epipharyngeal sclerite. Tentoropharyngeal sclerite from before the anterior half of the prothorax to almost the previous anterior half of metathorax, with two pairs of parastomal bars extending dorsally and ventrally along the hypopharyngeal sclerite, dorsal pair as long as 0.8 times the length of hypopharyngeal sclerite and ventral pair as long as 0.3 times the length of hypopharyngeal sclerite; dorsal bridge dorsally dark, extending anteriorly subsequently ¼ of hypopharyngeal sclerite; ocular spherical depression conspicuously located below the dorsal bridge; ventral cornu fused to the pharynx, forming a cavity that connects posteriorly with the esophagus and anteriorly with the cibarium; dorsal cornu 0.3 times shorter than the ventral cornu. Anterior spiracles, located dorsally in the latero-posterior half of pro-thorax, palmiform (Fig. 6a and b) variable across instars. Abdomen eight segmented, each (except I and VIII) in ventral view with two spinulose areas, anterior area with three transverse rows of spines, first anteriorly directed and second and third posteriorly directed, posterior area with only one transverse row of spines anteriorly directed; abdominal segment I, with two posteriorly directed anterior ventral rows, first with 36 (± 1) small spinules and second with 35 (± 2) papillae as spinules (Fig. 4c1), posterior row with 28 (± 2) spines directed above; abdominal segment VIII with two spinulose areas, anterior area with three transverse arranged rows of spines, first row with 34 (± 3) spines, anteriorly directed and the following two rows with 29 (± 2) and 43 (± 2) spines, posteriorly directed, posterior spinulose area with three transverse rows, the first continuous and the following two discontinuous: first row with 27 (± 4) spines, anteriorly directed and discontinuous rows with 8 (± 2) spines on each side, posteriorly directed (Fig. 3c3). Ventro-posterior rounded anal plate with a longitudinal slit in the middle; two dorso-posterior spiracular plates, located on abdominal protrusions, each with four processes as fractals (branchy structure) and spiracular openings that vary in number and shape on each instar (Fig. 6c and d).

**Figure 5.**
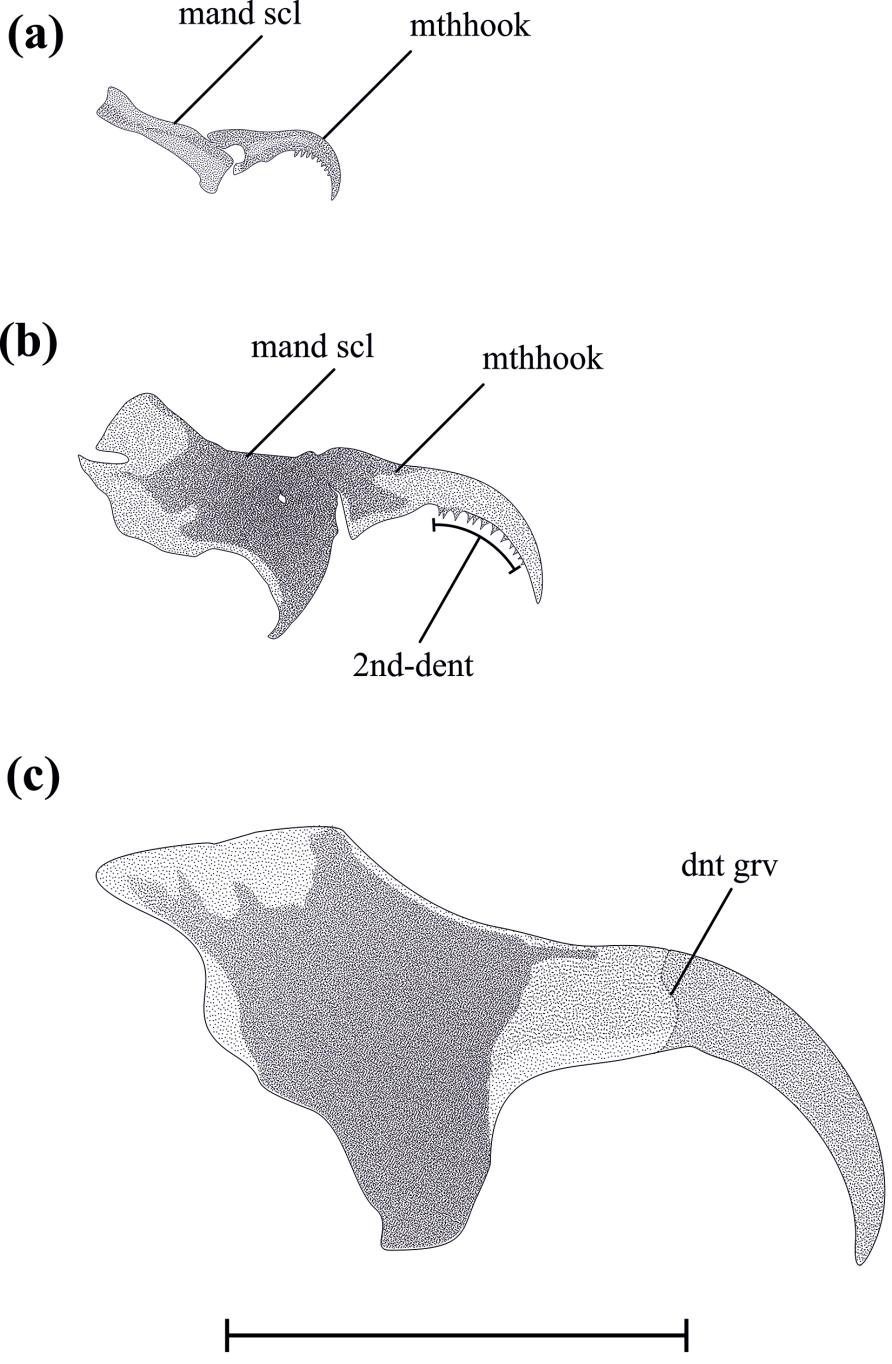
Mandibles of each larval instar. **a** L_1_ **b** L_2_ and **c** L_3_. dnt grv, dental groove; mand scl, mandibular sclerite; mthhook, mouthhook; 2nd-dent., secondary dentition. Scale bar: 1.5 mm.

**Figure 6.**
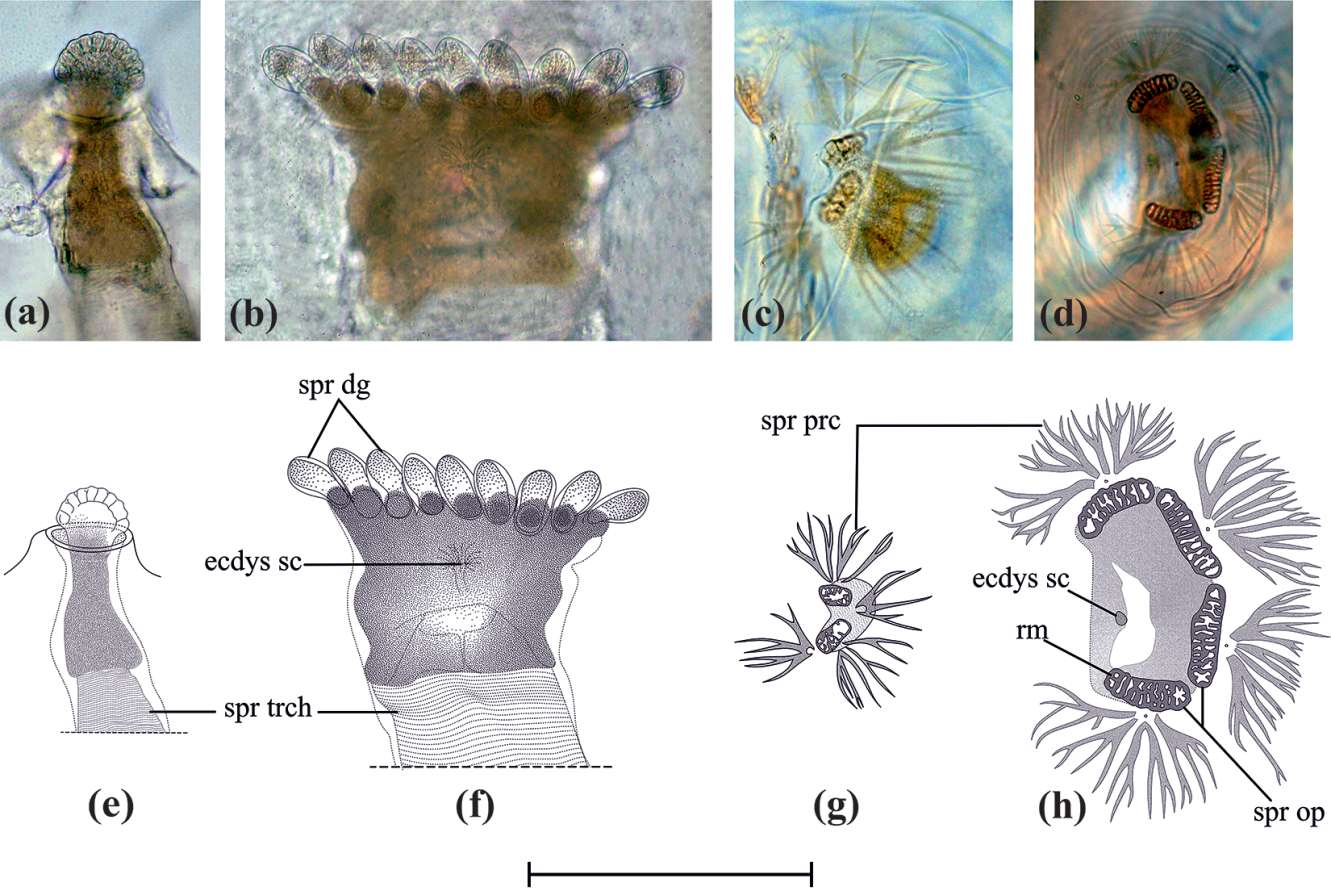
Spiracles of each larval instar. Anterior spiracle on L_2_ (**a, e**) and L_3_ (**b, f**). Posterior spiral on L_1_-L_2_ (**c, g**) and L_3_ (**d, h**). Scale bar of drawings 0.15 mm. ecdys sc, ecdysial scar; spr dg, spiracular digit; spr op, spiracular opening; spr prc, spiracular process; spr trch, spiracular trachea; rm, rime.

**Larval instar**

**L_1_.** From 1.22 to 2.52 mm (x = 1.76, n = 18) in length; antenna bi-segmented, apical segment oval; mandibular sclerite slightly sclerotized, elongated, three times longer than wide, dorsally articulated with the mouth hook, the latter with marked sclerotic outside and with 7–8 ventral teeth (Fig. 5a); anterior spiracles not observed (under light microscope); posterior spiracles with two semicircular spiracular openings (Fig. 6c) and four spiracular processes: two of them closely associated with spiracular openings, the other two free.

**Figure 7.**
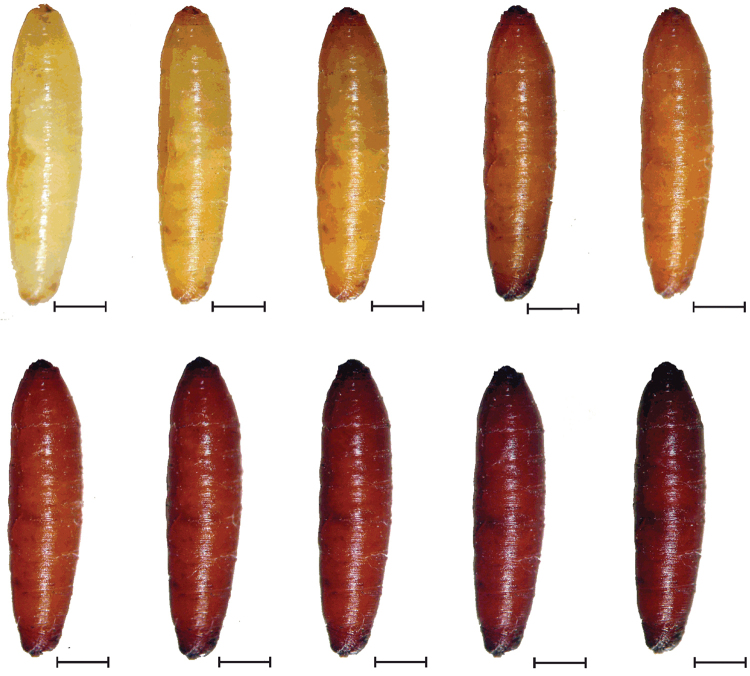
External coloration of Glyphidops (Oncopsia) flavifrons puparium, up to 3 hours after beginning cuticular exclerotization. From right to left: top row 0, 15, 30, 45, 60; bottom row 75, 90, 105, 135, and 180 minutes after beginning pupation. Scale bar: 1 mm.

**L_2_.** From 2.95 to 6.2 mm (x = 4.36; n = 22) in length; antenna with two antennomeres, distally oval; mandibular sclerite as long as 2.5 times wider, differentially sclerotized, antero-dorsally fused with mouth hook (Fig. 5b); ventral margin of mouth hook with 10 teeth; anterior spiracle 2.5 times longer than its greatest width, apical third visible as a small stump with 8–9 digital radiation and light interdigital recesses (Fig. 7e.); posterior spiracle as L_1_.

**L_3_.** From 6 to 11.16 mm (x = 9.17; n = 51) in length; tri-segmented antenna, second antennomere truncated, with sclerotized apical border, third antennomere reduced 0.3 times length of second like a papilla; mandible uniformly sclerotized, mandibular sclerite completely fused to mouth hook, reddish-brown, without ventral teeth mouth hook, showing a slight groove in basal ⅓ (Fig. 6c.); anterior spiracle 0.8 times as long as its greatest width, with 9–11 digits (Fig. 7b.), each on average 1.7 times longer than its greatest width, fully everted completely inside out, ecdysial conspicuous scar, mesal to digital projections (Fig. 7d); posterior spiracles with defined ecdysial scar, four spiracular openings elongated, mitochondrial-shaped (Fig. 6d), arranged semi-circularly to the scar, each opening with a spiracular process associated medially to the outer margin, each spiracular process radiates in a filamentous way to a small sclerotized point.

**Puparium**

Coarctate; 5.48–7.49 mm (x = 5.93; n = 15) in length, reddish brown (Fig. 3d and Fig. 7) with transverse striations or wrinkles that are more prominent on segments VII and VIII; anterior spiracles situated frontally (side view), with 9–11 sclerotized digits; posterior spiracles with four spiracular openings and a poorly defined spiracular process.

**Biology**

The body of each egg is buried in the substrate and the filament is spread over the surface. As the female began laying eggs (reaching packages up to 20 eggs, one egg at a time), it was observed that numerous filaments were emerging and radiating from the same point. Along this process, the male remains close to the female and the mating happen continuously between laid eggs. See Suppl. material 2.

Just moments before the hatching, the larva is observed moving the body and the head, rubbing its mandibles against the inner wall of the egg and finally thrusting the corium. The larva emerges from the anterior part of the egg by using one of the two longitudinally lateral hatching lines. See Suppl. material 3.

The pupation took between 150 and 175 minutes (n = 15) (Fig. 7a–k). The emergence of the imago was passed through a circular suture that ran from the anterior spiracles halfway to the first abdominal segment, separating two plates, one ventral and one dorsal, where the latter was completely separated from the puparium.

### Species comparison

Table 2 shows the main features found when comparing pre-imaginal stages described to date.

**Table 2.** Morphological comparison between immature stages of *Glyphidops flavifrons*, *Telostylinus lineolatus*, *Odontoloxozus longicornis* and *Odontoloxozus pachycericola*.

Feature	Glyphidops (Oncopsia) flavifrons	*Telostylinus lineolatus*	*Odontoloxozus longicornis*	*Odontoloxozus pachycericola*
Egg body length	1.0–1.24	-	0.89–1.09	-
Egg respiratory filament length	3.13–4.01	-	2.50–3.70	-
Anterior region of egg body with small elliptical tubercles band	Present	-	Absent	-
No. of antennomeres on L_3_	3	3	3	-
Ventral lobe on dorsal cornu	Absent	Absent	Absent	-
No. papillae on anterior spiracles on L_3_	9–11	8, 9 ‡	16 ‡, 17–19	13–15
No. of posterior spiracle openings on L_3_	4	4	4	4
Mandible composed on L_1_ and L_2_	Yes	-	Yes	-
Labial sclerite	Present	-	-	-
Epipharyngeal sclerite	Present	-	-	-
Hypophr. scl. Length on L_3_ (mm)	0.236–0.242	-	0.27–0.31	-
Body length on L_3_ (mm)	6–11.16	5.9–8.1	8.77–13.28	-
Puparium length (mm)	5.48–7.49	4.8–6.3	5.8–8.75	.

(-) Unknown, (‡) Usually

## Discussion

According to the Brooks-Dyar rule (Dyar 1890, Crosby 1973, Hutchinson and Tongring 1984) the growth rate of one or more sclerotized structures increases at a geometric rate throughout the larval stages. Over the years this rule has become an indispensable tool for the description and establishment of the larval stages for many holometabolous insect species. For Muscomorpha, research focused on the variation of hypopharyngeal sclerites or mandibles (Petitt 1990) and showed that the cephalic tagma has lost its outer sclerotization and is reduced to a membranous area. As shown in figures 1a and 1c, two significant leaps in the individual measurements of each structure were observed, with a growth ratio of 1.31 (L_1_ to L_2_) and 1.84 (L_2_ to L_3_) for hypopharyngeal sclerite length, and 5.44 (L_1_-L_2_) and 3.26 (L_2_ to L_3_) in the mandibular area. Additionally, it was possible to determine each larval stage through tracking the changes of external morphological features, such as the rise of the anterior spiracles on L_2_ and its noticeable modification when entering L_3_, as well as the increase of spiracular posterior openings (two to four) when transitioning from L_2_ to L_3_, and finally the significant changes of the mandibular sclerotization and loss of the mouth hook’s ventral teeth, throughout each of the instars. Even though the anterior spiracle was not distinct on L_1_ under the microscope light, some authors have found evidence of the presence of this on several Schizophora species using the scanning electron microscope (Kitching 1976, Grzywacz 2012). It is possible that an anterior spiracle exists in Glyphidops (Oncopsia) flavifrons L_1_, however additional studies are required.

On the larval descriptions of Diopsoidea’s families (sister group of Nerioidea *sensu* McAlpine), only two allusions to the labial and the epipharyngeal sclerite have been done. The first one on *Sphyracephala brevicornis* (Say) says that both features were undeveloped (Lavigne 1962). The second one by Foote (1970), on *Tanypeza longimana* Fallén showed a broadly V-shaped ligulate sclerite (= labial sclerite, Foote and Teskey 1991) immediately anterior to the hypostomal plate (= hypopharyngeal sclerite, Teskey 1981). The shape and position of the “ligulate sclerite” suggests that the labial sclerite found in Glyphidops (Oncopsia) flavifrons, represents a homologous structure.

Foote and Teskey (1991) undertook a morphological review of Diptera larvae, they did not find specific features to differentiate Neriidae larvae from other closely related saprophagous families, but proposed the four elliptical openings surrounding the ecdysial scar in the posterior spiracles as a potentially strong diagnostic feature. Our study confirms their proposal and additionally reports two new features for the family: the presence of the epipharyngeal sclerite and the labial sclerite. These two novel features are also new at the superfamily level since neither of them have been reported in Cypselosomatidae, or Micropezidae larval descriptions (Berg 1947, Bohart and Gressitt 1951, Olsen and Ryckman 1963, McAlpine 1966, Wallace 1969, Teskey 1972, Foote and Teskey 1991, Mangan and Baldwin 1986, Barnes 2015).

The morphological characters observed in Glyphidops (Oncopsia) flavifrons immature stages indicate that both adult and larval stages of nerioids flies retain plesiomorphic features, such as larva with filter apparatus for particle feeding, mandibles separate, parastomal bars present and dorsal cornua with a window. Nevertheless, there are some autapomophies in Neriidae, such as eggs with longitudinal dorsal hatching seam; these are not in the ground plan of Acalyptrata. The monophyletic group Neriidae+Cypselosomatidae was initially proposed by McAlpine (1989) supported by seven synapomorphies of adults flies. Therefore, we propose that the four elliptical openings, surrounding the ecdysial scar in the posterior spiracles of L_3_, serve as a synapomorphy of the larval stage, since this condition is only found within these two families of Nerioidea and it is not known to appear in any other Acalyptrate taxon (Teskey 1981, Foote and Teskey 1991, Borkent and Rotheray 2009).

Olsen and Ryckman (1963) stated that *Odontolozoxus longicornis* could be differentiated from *Telostylinus lineolatus* by the number of anterior spiracle digits. Likewise, Mangan and Baldwin (1986) found that the same feature allowed the separation of *Odontoloxozus longicornis* from *Odontoloxozus pachycericola*. Glyphidops (Oncopsia) flavifrons supports the use of the number of anterior spiracle digits as a consistent feature to separate the four species (Table 2). Nonetheless, the overlap between number of digits may generate difficulties in the future, thereby a further morphometric study (of gradual growth of the hypopharyngeal sclerite and mandibles) is recommended to determine its potential usefulness as a diagnostic character to differentiate larval neriids.

## Supplementary materials

Supplementary material 1 Cluster analysis of mandibular area and hypopharyngeal sclerite Data type: TIF file Explanation note: Cluster analysis of mandibular area and hypopharyngeal sclerite length across time supports the concept of three larval instar (similarity indexes between 95% and 100%). (a) body length of the larva, (b) mandibular area and (c) length of hypopharyngeal sclerite. Each color represents a larval instar (Ln). This dataset is made available under the Open Database License (http://opendatacommons.org/licenses/odbl/1.0/). The Open Database License (ODbL) is a license agreement intended to allow users to freely share, modify, and use this Dataset while maintaining this same freedom for others, provided that the original source and author(s) are credited.Andrés Felipe Vinasco Mondragón, Nancy Soraya Carrejo Gironza

Supplementary material 2 Mating and oviposition process of Glyphidops (Oncopsia) flavifrons Data type: Video mp4 file Explanation note: The body of each egg is buried in the substrate and the filament is spread over the surface. As the female began laying eggs (reaching packages up to 20 eggs, one egg at a time), it was observed that numerous filaments were emerging and radiating from the same point. Along this process, the male remains close to the female and the mating happen continuously between laid eggs. This dataset is made available under the Open Database License (http://opendatacommons.org/licenses/odbl/1.0/). The Open Database License (ODbL) is a license agreement intended to allow users to freely share, modify, and use this Dataset while maintaining this same freedom for others, provided that the original source and author(s) are credited.Andrés Felipe Vinasco Mondragón, Nancy Soraya Carrejo Gironza

Supplementary material 3 Egg hatching of Glyphidops (Oncopsia) flavifrons Data type: Video mp4 file Explanation note: Moments before the hatching, the larva is observed moving the body and the head, rubbing its mandibles against the inner wall of the egg and finally thrusting the corium. The larva emerges from the anterior part of the egg by using one of the two longitudinally lateral hatching lines. This dataset is made available under the Open Database License (http://opendatacommons.org/licenses/odbl/1.0/). The Open Database License (ODbL) is a license agreement intended to allow users to freely share, modify, and use this Dataset while maintaining this same freedom for others, provided that the original source and author(s) are credited.Andrés Felipe Vinasco Mondragón, Nancy Soraya Carrejo Gironza
