# Supplementary figures and images for: Morphology and development rate of the immature stages of Glyphidops (Oncopsia) flavifrons (Bigot, 1886) (Diptera, Neriidae) under natural conditions
Source: Zookeys. 2016 Jul 6;(603):141–59. doi: 10.3897/zookeys.603.7355 (PMC4978005; doi:10.3897/zookeys.603.7355)

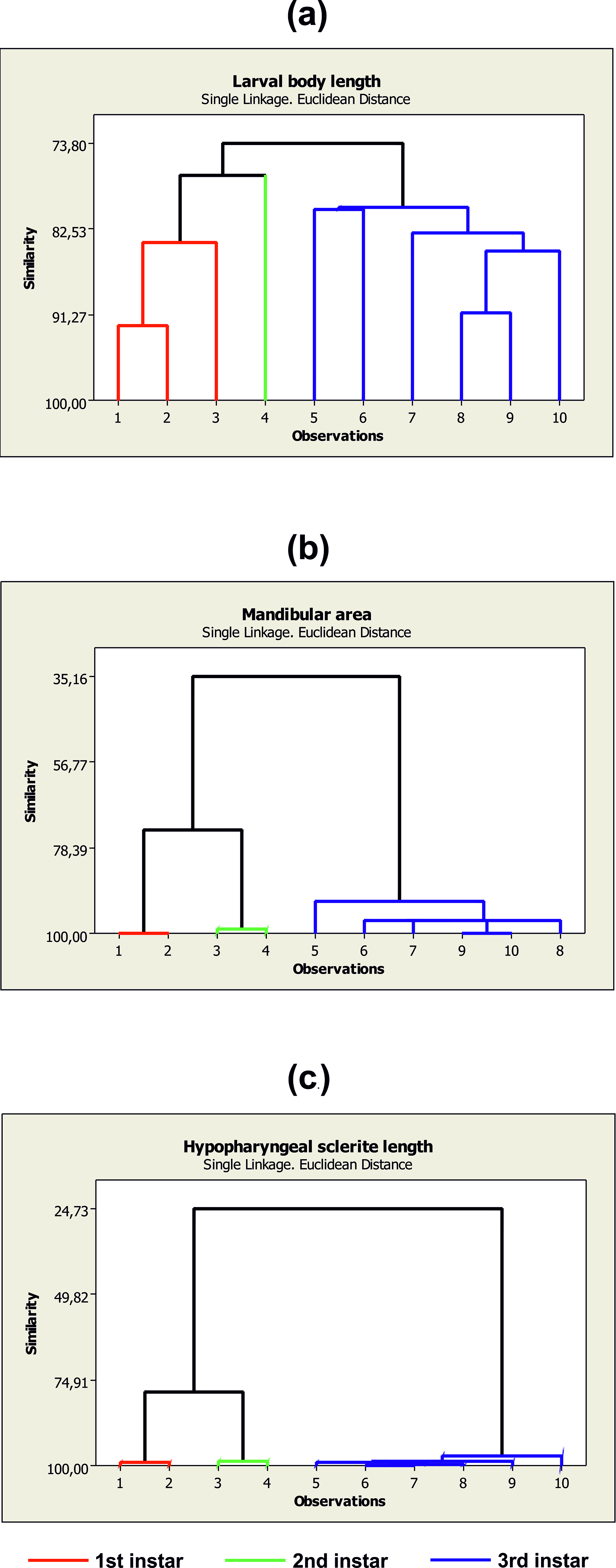

Supplement: Supplementary material 1 — Cluster analysis of mandibular area and hypopharyngeal sclerite [file zookeys-603-141-s001.tif]
